# Phenotyping trisomies 13 and 18 and CHARGE syndrome in fetal MRI—a proposed phenome-based, morphological disease severity score, and network medicine analysis

**DOI:** 10.1007/s00330-026-12503-w

**Published:** 2026-04-29

**Authors:** Marlene Stuempflen, Michael Weber, Ursula Schwarz-Nemec, Anke Scharrer, Hui Shi, Victor U. Schmidbauer, Patric Kienast, Katharina Goeral, Guelen Yerlikaya-Schatten, Gregor Kasprian, Daniela Prayer

**Affiliations:** 1 Department of Biomedical Imaging and Image-guided Therapy, Medical University of Vienna, Vienna, Austria; 2 Department of Pathology, Medical University of Vienna, Vienna, Austria; 3 Department of Radiology, Zhu Jiang Hospital, Southern Medical University, Guangzhou, China; 4 Division of Neonatology, Intensive Care and Neuropediatrics, Department of Pediatrics and Adolescent Medicine, Comprehensive Center for Pediatrics, Medical University of Vienna, Vienna, Austria; 5 Department of Obstetrics and Gynaecology, Division of Obstetrics and Feto-Maternal Medicine, Medical University of Vienna, Vienna, Austria

**Keywords:** CHARGE syndrome, Magnetic resonance imaging, Trisomy 13, Trisomy 18, Fetal MRI

## Abstract

**Objectives:**

CHARGE syndrome (CS) and trisomy 13 (T13) and 18 (T18) are heterogeneous diseases with overlapping morphological features. Historically, T13 and T18 were deemed incompatible with life. Recently, numerous studies have reported prolonged survival for some affected patients. Consequently, the question of individual counseling has arisen. This study aimed to analyze the fetal MRI-based phenome of CS, T13, and T18.

**Materials and methods:**

Fetal MRI-based phenotyping was conducted, and a morphological disease severity score that assessed 16 anatomical regions was proposed. Furthermore, a co-occurrence analysis was generated to visualize the overlapping and differentiating features of CS, T13, and T18.

**Results:**

Forty-eight fetuses who underwent fifty-seven fetal MRI scans were analyzed. Disease severity scores ranged from 1-25 (mean 12.7) and highlighted heterogeneous disease manifestations among investigated patients. In the co-occurrence analysis the T13 network showed the highest complexity.

**Conclusion:**

Considering recent trends towards a change in management from mostly palliative to therapeutic care for patients with CS, T13, and T18, care providers face challenging decisions regarding management. The proposed preliminary MRI-based phenotyping score and the provided phenome visualization aim to aid physicians in counseling and choosing appropriate management plans. Future studies will be necessary to correlate prenatal imaging findings to outcome data in larger patient collectives.

**Key Points:**

***Question*** *What are the phenotypical presentations of CHARGE syndrome, trisomy 13, and trisomy 18 in fetal MRI and can prenatal MRI findings help clinicians in predicting postnatal outcomes?*

***Findings**** MRI phenomes were visualized in co-occurrence networks, and a preliminary disease severity score was proposed, based on available outcome data, to aid in risk stratification*.

***Clinical relevance**** Recent trends in management, a shift from mostly palliative to therapeutic care for affected patients, have challenged clinicians. The provided phenome visualization of these heterogeneous diseases and the proposed disease severity score may aid physicians in counseling and selecting appropriate pregnancy management*.

**Graphical Abstract:**

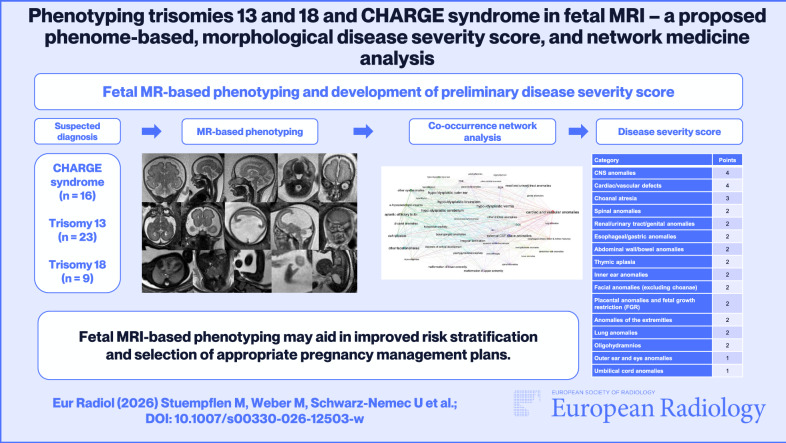

## Introduction

Trisomy 13 (T13) and 18 (T18) were long considered fatal diseases with a life expectancy of less than a year [1, 2]. In recent years, multiple studies have reported prolonged survival with the implementation of advanced therapies and life-prolonging interventions more commonly offered [1, 3–6]. Due to modern approaches of choosing management plans based on phenotyping of genetically determined conditions, some patients were reported to have survived longer than 10 years [2].

CS is a rare genetic disorder with a prevalence of 0.1 in 100,000 births [7], and 10-year survival rates were estimated between 70%–87% [8, 9]. In contrast, a large nationwide Korean study determined the prevalence of T18 to be as high as 2.7–6.4 per 100,000 births with a median survival of 127 days [10]. They identified a 1-year survival rate of 63.2%, and the survival rate at first hospital admission of patients, who did and did not have congenital heart disease, was 58.3% and 94.1%, respectively [10]. Furthermore, it was determined that patients with congenital heart defects, who underwent surgical or catheter intervention, had longer survival times than those who did not [10]. Another Korean study determined the prevalence of T13 to be smaller, with 0.02 per 100,000 births, responsible for nearly 1% of all birth defects in the population [11]. Another large European study quantified one-year survival rates as 17% (T13) versus 13% (T18) and 10-year survival as 11% (T13) versus 8% (T18), respectively [2].

CHARGE syndrome (CS) presents with a wide spectrum of symptoms, including choanal atresia, ocular, cardiac, genital, and ear anomalies caused by mutations within the CHD7 gene. However, recent studies have shown the importance of mutational screening of CHD7 even in patients who display clinical features of CS despite having previously been diagnosed with other syndromes, highlighting the fact that CHARGE should be considered a disease spectrum with overlapping structural anomalies even without a confirmed CHD7 mutation [12]. Furthermore, continuous identification of novel mutations in confirmed cases with CS, as identified in a recent Korean study conducted by Sohn et al, highlights the need for continuously updated screening panels in the identification of patients with CS [13].

T13, T18, and CS have overlapping pathomorphological features, including cardiac, renal, esophageal, midfacial, ocular, and central nervous system (CNS) anomalies and altered development of the extremities [12, 14]. This may cause diagnostic uncertainty in the absence of prior genetic testing or, specifically regarding CHARGE syndrome, negative genetic testing.

In light of recent changes in healthcare policies and legislative regulation regarding abortion laws, evidence-based counseling needs to adapt accordingly and provide individualized treatment depending on phenotypical presentation [4, 15]. As genome-wide genotyping has become more readily available within the last two decades, the importance of differentiating between the genotype and the often much more heterogenous phenotype must be considered clinically. Thus, relying on genetics alone may be insufficient to estimate postnatal disease burden.

The aim of this study was twofold: first, to describe the phenotype of CS and trisomy 13 and 18, and second, to extract morphological features within these phenotypes that allow for a respective prognosis. Following a phenotypical assessment, a network medicine analysis was conducted. Furthermore, a phenome-based disease severity score that assessed the extent of the observed morphological changes was proposed to provide an estimation of disease burden and to aid clinicians in choosing appropriate management plans.

## Materials and methods

### Study design and patients

This study was approved by the institutional ethics board (EK number 1547/2021), and written informed consent was obtained from all patients. Women with singleton pregnancies undergoing fetal MRI at a tertiary care center from January 2004 until November 2023 were investigated retrospectively. All MRI examinations were clinically indicated and referred by fetal medicine specialists. Institutional patient records were screened, and women carrying fetuses with genetically confirmed cases of T13, T18, and CS and available fetal or post-mortem MRI were identified, and maternal medical history was extracted. Following the initial identification of fetuses, inclusion and exclusion criteria were applied: fetuses were analyzed if their gestational age had been determined by first-trimester ultrasound, a detailed second-trimester ultrasound scan had been conducted, and high-quality fetal MRI data were available. Missing data were completed using available patient records. Cases with incomplete data after screening of institutional patient records were excluded.

### Fetal MRI

Fetal MRI was conducted using 1.5 T and 3 T magnets (Philips Ingenia/Intera and Philips Achieva). Examinations were performed supine or, if necessary, in the left recumbent position within 45 min. Neither MRI contrast medium nor sedation was applied. Due to the retrospective nature of this study and the long inclusion interval of patients, various imaging guidelines regarding fetal MRI were applied. However, all MRI scans included in this study were checked for compliance with the 2023 International Society of Ultrasound in Obstetrics & Gynecology Practice Guidelines [16]. Fetal brain imaging included T2-weighted sequences in three orthogonal planes of the fetal head (slice thickness 2.0–4.5 mm, field of view = 200–230 mm, echo time = 100–140 ms, in-plane resolution 0.62/0.62–1.17/1.17 mm).

### Phenotypical fetal assessment

MRI data of included fetuses were reviewed by two experienced neuroradiologists simultaneously, who were blinded to clinical history, including ultrasound report diagnosis and genetic diagnosis, in consensus (D.P.—35 years of experience in fetal MRI, M.S.—five years of experience in fetal MRI). Morphological analysis of investigated fetuses was performed and included the detection of anomalies in 22 anatomical regions: cranial nerves I and II; cortical development; brain symmetry; presence of holoprosencephaly; cerebral midline structures; cerebrospinal fluid (CSF) spaces; acquired intraventricular and/or parenchymal lesions of the brain; brainstem and aqueduct; cerebellum and vermis; head/skull (including facial; jaw, palate; tongue anomalies and choanal atresia); eyes; ears; cardiovascular system; lung; neck; esophagus and organs of the upper quadrants; bowels and abdominal wall; urinary and reproductive system; skeleton; placenta and presence of fetal growth restriction (FGR); and amniotic fluid. Ventriculomegaly was defined as a lateral ventricle atrial diameter of greater than 10 mm. Hypo- and hypertelorism were defined by an interocular diameter smaller than and greater than the ocular diameter, respectively. Cerebellar diameter was measured in the axial or coronal plane and compared to reference values as provided by Prayer et al [17]. Regarding the presence of cardiac defects, the assessment was focused on reported ultrasound findings, as more subtle cardiac defects are often not visible on fetal MRI. Percentages of detected anomalies for each disease group were calculated.

### Statistical analysis and co-occurrence network

Visualization of patient selection was provided in a flowchart using the software MindManager (version 23.0).

Nominal data are described as absolute frequencies (percentages). Mean +/− SD, as well as (minimum and maximum) were used to describe average severity scores per group. Normal distribution was tested using Shapiro–Wilks test as well as visually checked by QQ-plots. P-values equal to or below 0.05 were considered statistically significant. Statistical analysis was performed using the software IBM SPSS Statistics for Windows (version 28).

To visually assess the phenotypically differentiating and overlapping features of CS, T13, and T18, a technique was used similar to that applied by Baldassano et al [18]: three co-occurrence matrices for each investigated group were generated using MATLAB (version R2021b) based on CS, T13, and T18 cases. Each matrix was generated to extract co-occurrence patterns of morphological findings of fetal MRI scans as documented by two authors in consensus (D.P.—35 years of experience in fetal MRI, M.S.—five years of experience in fetal MRI). Subsequently, network co-occurrence graphs were generated using the software Gephi (version 0.10.1) (H.S., M.S) [19].

### Disease severity score

To allow for faster and easier risk stratification, a simplified disease severity score was calculated based on the detection of anomalies summarized in 16 categories (Table 1): CNS; cardiac/vascular; choanal; pulmonary; spinal; genital; renal/urinary tract; esophageal/gastric; abdominal wall/bowel; thymic; facial; eye/outer ear; inner ear; placental (including FGR); umbilical anomalies; malformation of the extremities; and the presence of oligohydramnios.

**Table 1:** Proposed morphological disease severity score

Category	Points
CNS anomalies	4
Cardiac/vascular defects	4
Choanal atresia	3
Spinal anomalies	2
Renal/urinary tract/genital anomalies	2
Esophageal/gastric anomalies	2
Abdominal wall/bowel anomalies	2
Thymic aplasia	2
Inner ear anomalies	2
Facial anomalies (excluding choanae)	2
Placental anomalies and fetal growth restriction (FGR)	2
Anomalies of the extremities	2
Lung anomalies	2
Oligohydramnios	2
Outer ear and eye anomalies	1
Umbilical cord anomalies	1

The presence of detected anomalies within each category was graded with one to four points, with higher scores indicating a worse outcome. Due to incomplete outcome data in the investigated patient collective, the score for each category was based on previously published data, as indicated in the Discussion section of this manuscript. The grading was agreed upon by the authors of this study following a Delphi-type consensus technique [20]. Lastly, institutional patient records were screened to identify available outcome data of investigated fetuses.

## Results

### Patients and demographics

**Fig. 1:**
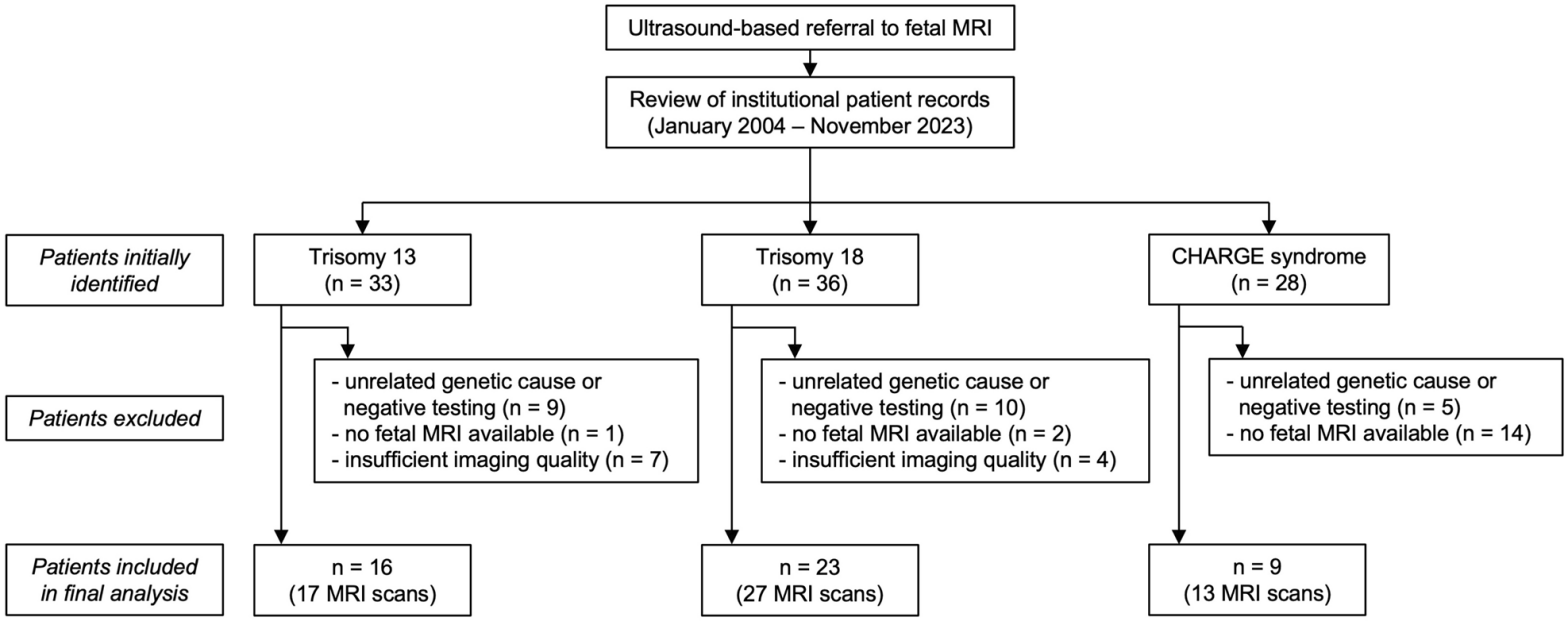
Patient identification flow-chart

The patient identification and selection processes are summarized in Fig. 1. Following initial review of institutional patient records, a total of 96 fetuses were identified: 28 with a suspected diagnosis of CS, 33 with T13, and 36 with T18. During patient assessment, a total of 47 fetuses had to be excluded for the following reasons, among them 19 fetuses with suspected CS (20 scans), 17 T13 (undergoing 20 MRI scans), and 13 T18 (13 scans): confirmatory genetic testing for other, unrelated diseases or inconclusive follow-up (26 cases); no available fetal or post-mortem MRI (only postnatal imaging available) (17 cases); and insufficient fetal MRI imaging quality (eleven cases). Three fetuses suspected of suffering from CS had inconclusive and incomplete genetic testing: one fetus did not undergo genetic testing specifically for CS, with unremarkable results following fluorescence in situ hybridization (FISH) and a single-nucleotide polymorphism (SNP) array. Two other fetuses (undergoing three fetal MRI scans) underwent an unremarkable karyotype and array CHG testing, and their mothers did not consent to further genetic testing specifically for CS. All three children are still currently clinically assigned as patients with CS and were thus included in this study. Thus, a total of 48 fetuses (42 fetal and 15 post-mortem MRI scans) were included in the final analysis of this study: nine suspected of suffering from CS (undergoing thirteen MRI scans), 16 with T13 (17 scans), and 23 with T18 (27 scans).

A demographic overview of the investigated fetuses, including sample size, mean gestational and maternal age, and fetal sex, is provided in Table 2.

**Table 2:** Demographics

	CS	T13	T18
Sample size	9 (13)	16 (17)	23 (27)
Number of MRI scans (in utero + post mortem)	13 + 0	13 + 4	16 + 11
Gestational age (weeks)	26.4 + /− 4.6	26.0 + /− 6.1	25.5 + /− 7.3
Maternal age (years)	33.2 + /− 6.1	30.8 + /− 9.7	36.9 + /− 6.0
Fetal sex (female)	4 (44.4%)	8 (50.0%)	16 (69.6%)

Sample size given in number of fetuses (number of scans), ages given in mean +/− standard deviation

*CS* CHARGE syndrome, *T13* trisomy 13, *T18* trisomy 18

### Phenotypical fetal assessment

Quantitative statistical analysis of morphological features of each group was conducted and is summarized in Table 3. The most frequently observed anomalies are described in Table 4.

**Table 3:** Phenotypical assessment

Anatomical structure	CS	T13	T18	Overall
Optic tracts/chiasma	0 (0%)	6 (35.3%)	2 (7.4%)	8 (14.0%)
Olfactory bulbi	7 (53.8%)	10 (58.8%)	8 (29.6%)	25 (43.9%)
Sulcation & gyration	2 (15.4%)	10 (58.8%)	6 (22.2%)	18 (31.6%)
Lamination	3 (23.1%)	9 (52.9%)	4 (14.8%)	16 (28.1%)
Brain symmetry	5 (38.5%)	10 (58.8%)	4 (14.8%)	19 (33.3%)
Basal ganglia	0 (0%)	7 (41.2%)	3 (11.1%)	10 (17.5%)
Holoprosencephaly	2 (15.4%)	7 (41.2%)	4 (14.8%)	13 (22.8%)
Cerebral midline structures	5 (38.5%)	12 (70.6%)	11 (40.7%)	28 (49.1%)
Lateral ventricles	4 (30.8%)	9 (52.9%)	11 (40.7%)	25 (43.9%)
Ventricle lining	2 (15.4%)	2 (11.8%)	2 (7.4%)	6 (10.5%)
CSF spaces	2 (15.4%)	6 (35.3%)	5 (18.5%)	13 (22.8%)
Fossa posterior	1 (7.7%)	5 (29.4%)	10 (37.0%)	16 (28.1%)
Intraventricular lesions	0 (0%)	1 (5.9%)	2 (7.4%)	3 (5.3%)
Cerebral parenchymal lesions	3 (23.1%)	2 (11.8%)	0 (0%)	5 (8.8%)
Brainstem	9 (69.2%)	12 (70.6%)	17 (63.0%)	38 (66.7%)
Aqueduct	1 (7.7%)	2 (11.8%)	2 (7.4%)	5 (8.8%)
Cerebellum	9 (69.2%)	11 (64.7%)	16 (59.3%)	36 (6.2%)
Vermis	6 (46.2%)	12 (70.6%)	12 (44.4%)	30 (52.6%)
Skull	1 (7.7%)	5 (29.4%)	6 (22.2%)	12 (21.5%)
Midface anomalies	3 (23.1%)	7 (41.2%)	3 (11.1%)	13 (22.8%)
Chin/jaw anomalies	1 (7.7%)	4 (23.5%)	8 (29.6%)	13 (22.8%)
Cleft	5 (38.5%)	8 (47.1%)	3 (11.1%)	16 (28.1%)
Tongue	2 (15.4%)	0 (0%)	0 (0%)	2 (3.5%)
Choanal atresia	4 (30.8%)	5 (29.4%)	5 (18.5%)	14 (24.6%)
Eyes	9 (69.2%)	11 (64.7%)	17 (63.0%)	37 (64.9%)
Inner ear	2 (15.4%)	2 (11.8%)	0 (0%)	4 (7.0%)
Outer ear	5 (38.5%)	11 (64.7%)	4 (14.8%)	21 (36.8%)
Vitium cordis	7 (53.8%)	11 (64.7%)	22 (81.5%)	41 (71.9%)
Vascular structures	3 (23.1%)	2 (11.8%)	1 (5.9%)	7 (12.3%)
Lung	0 (0%)	1 (5.9%)	1 (5.9%)	2 (3.5%)
Neck	5 (38.5%)	2 (11.8%)	4 (14.8%)	11 (19.3%)
Thymus	2 (15.4%)	2 (11.8%)	0 (0%)	4 (7.0%)
Esophagus/stomach	5 (38.5%)	3 (17.6%)	13 (76.5%)	21 (36.8%)
Liver/spleen/gallbladder	1 (7.7%)	2 (11.8%)	3 (11.1%)	6 (10.5%)
Bowels	1 (7.7%)	1 (5.9%)	2 (7.4%)	4 (7.0%)
Abdominal wall	0 (0%)	3 (17.6%)	6 (22.2%)	9 (15.8%)
Kidneys	3 (23.1%)	8 (47.1%)	4 (14.8%)	16 (28.1%)
Urinary tract and suprarenal glands	0 (0%)	3 (17.6%)	1 (5.9%)	4 (7.0%)
Gonads	0 (0%)	2 (11.8%)	2 (7.4%)	4 (7.0%)
Upper extremity	2 (15.4%)	8 (47.1%)	9 (52.9%)	20 (35.1%)
Lower extremity	0 (0%)	7 (41.2%)	4 (14.8%)	11 (19.3%)
Spine	0 (0%)	2 (11.8%)	0 (0%)	2 (3.5%)
Umbilical cord	1 (7.7%)	7 (41.2%)	5 (18.5%)	14 (24.6%)
Fetal growth restriction (FGR)	0 (0%)	3 (17.6%)	10 (37.0%)	13 (22.8%)
Placenta	1 (7.7%)	4 (23.5%)	4 (14.8%)	9 (15.8%)
Amniotic fluid	4 (30.8%)	3 (17.6%)	9 (52.9%)	16 (28.1%)

Summary of patients with anomalies in the included anatomical structures, given in the number of patients (percentage of patients with this diagnosis from all groups). Percentages may vary between anatomical structures, as not all structures were differentiable in every fetus, depending on neurodevelopmental progress/gestational age of the fetus at the timepoint of MRI and consecutive assessability on fetal MRI

*CS* CHARGE syndrome, *T13* trisomy 13, *T18* trisomy 18

**Table 4:** Summary of the most frequently observed anomalies among all investigated groups

Most frequently observed anomalies	CS	T13	T18
Cardiac/vascular anomalies	10 (76.9)	13 (76.5)	14 (50.0)
Hypo-/dysplastic brainstem	9 (69.2)	12 (70.6)	17 (60.7)
Hypo-/dysplastic vermis	6 (46.2)	12 (70.6)	14 (50)
Hypo-/dysplastic cerebellum	9 (69.2)	11 (64.7)	16 (57.1)
Hypo-/dysplastic outer ear	5 (38.5)	11 (64.7)	5 (17.9)
Aplastic olfactory bulbi	7 (53.8)	10 (58.8)	8 (28.6)
External CSF space anomalies	3 (23.1)	10 (58.8)	12 (43.0)
Other facial anomalies	4 (30.8)	9 (52.9)	9 (32.1)
Renal/urinary tract	3 (23.1)	9 (52.9)	5 (17.9)
Esophageal atresia (in-/direct features)	5 (38.5)	3 (17.6)	13 (46.4)
Hypertelorism	7 (53.8)	1 (5.9)	2 (7.1)
Hypotelorism	1 (7.7)	4 (23.5)	15 (53.6)

Given the number of MRI scans with observed anomalies (percentage for each group)

*CS* CHARGE syndrome, *T13* trisomy 13, *T18* trisomy 18

All three groups presented with high rates of cardiovascular and CNS anomalies, specifically affecting the brainstem, vermis, cerebellum, external CSF spaces, and the olfactory bulbi. Abnormal eye distance (i.e., hypo- and hypertelorism) seemed to contrarily affect trisomies and CS.

**Fig. 2:**
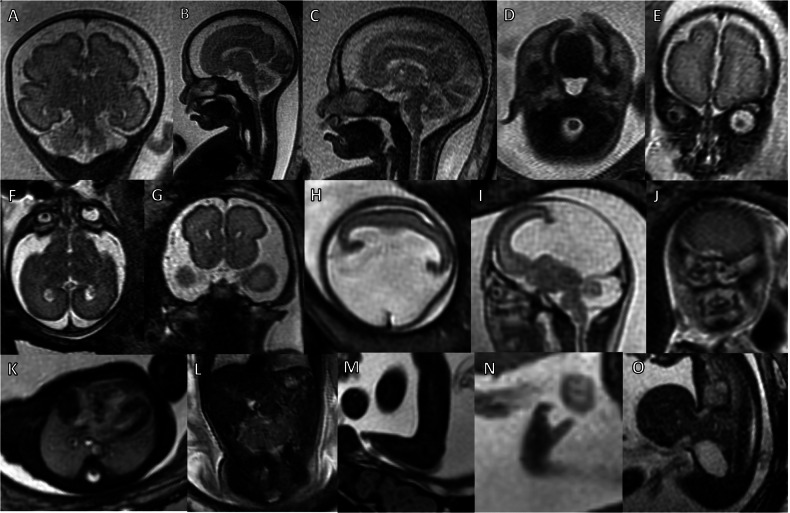
MR phenotyping CHARGE syndrome and trisomy 13 and 18. First row (**A**–**E**): CHARGE syndrome A, widened external CSF spaces (29 + 1 weeks gestational age [GW], T2-weighted MRI); **B** esophageal atresia with cervical pouch sign (29 + 1 GW, T2); **C** + **D** cleft lip and palate (29 + 1 GW, T2); **E** aplastic olfactory bulbi (29 + 5 GW, T2). Second row (**F**–**J**): trisomy 13 F, microphthalmia (28 + 3 GW, T2); G, microtia (28 + 3, T2); **H**–**J**, alobar holoprosencephaly with microphthalmia and choanal atresia (21 + 5 GW, T2). Third row (**K**–**P**): trisomy 18. **K** ventricular septum defect (32 + 0 GW, steady-state free precessing MRI-SSFP); **L** horseshoe kidney (32 + 0 GW, SSFP); **M** rocker-bottom feet (32 + 1 GW, SSFP); **N** ectrodactyly (23 + 3 GW, T2); **O** omphalocele (29 + 1 GW, SSFP)

A detailed overview of all included patients is provided in Table S1 in the supplementary material, with a summary of all observed anomalies within each anatomical region in Table S2. Visualization of selected structural anomalies identified in fetal MRI is provided in Fig. 2.

### Co-occurrence matrix

**Fig. 3:**
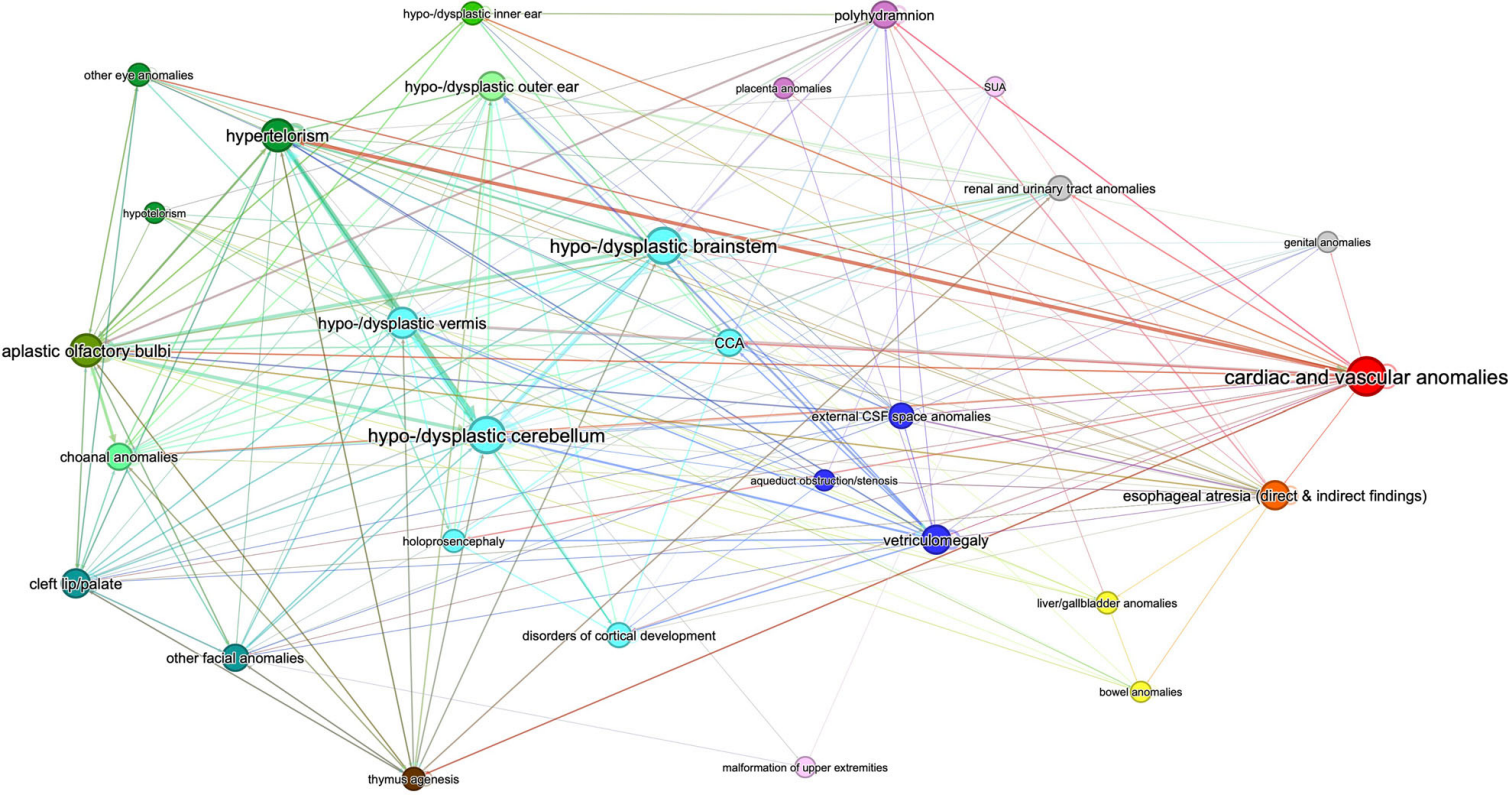
Co-occurrence network of patients with CHARGE syndrome

**Fig. 4:**
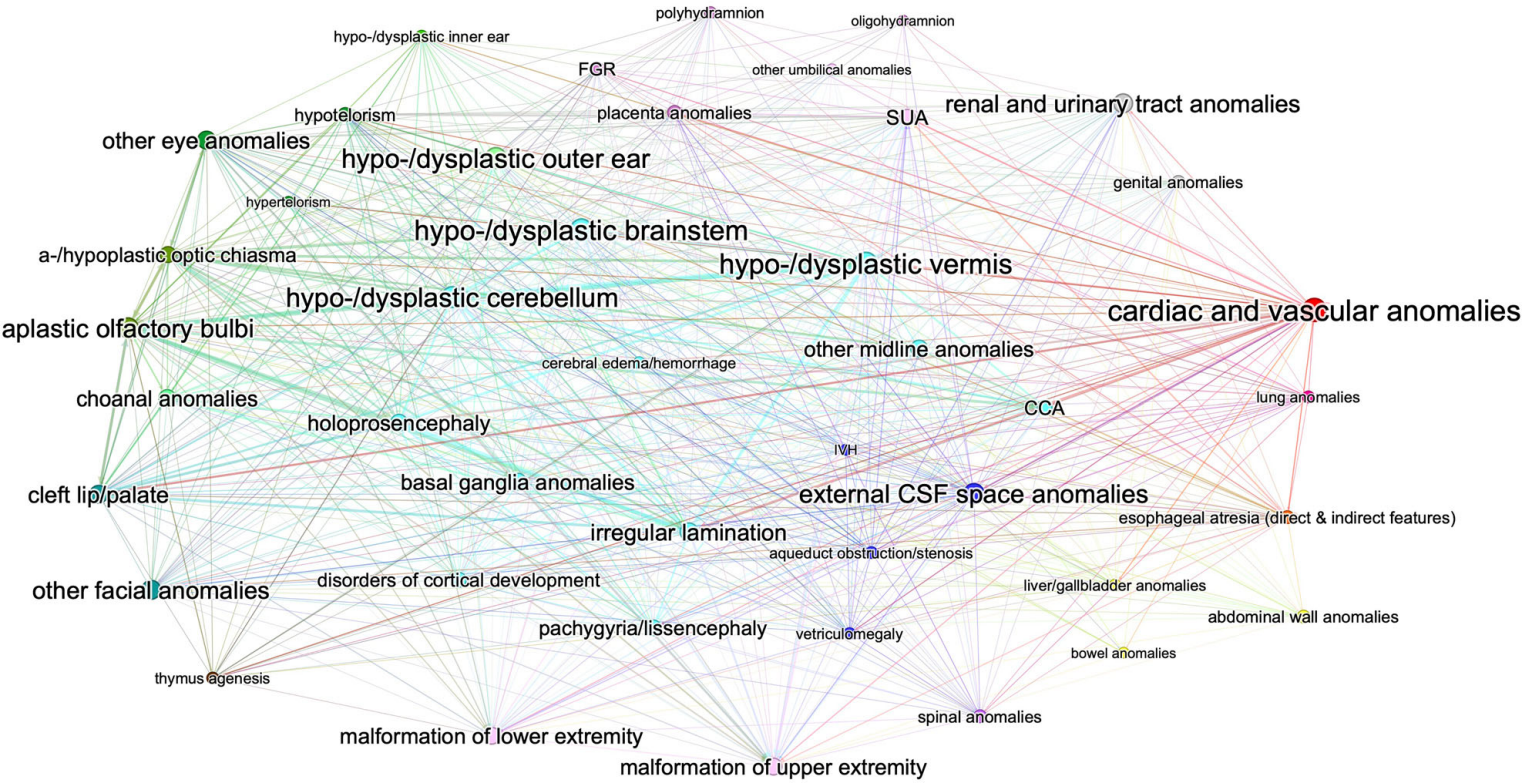
Co-occurrence network of patients with trisomy 13

**Fig. 5:**
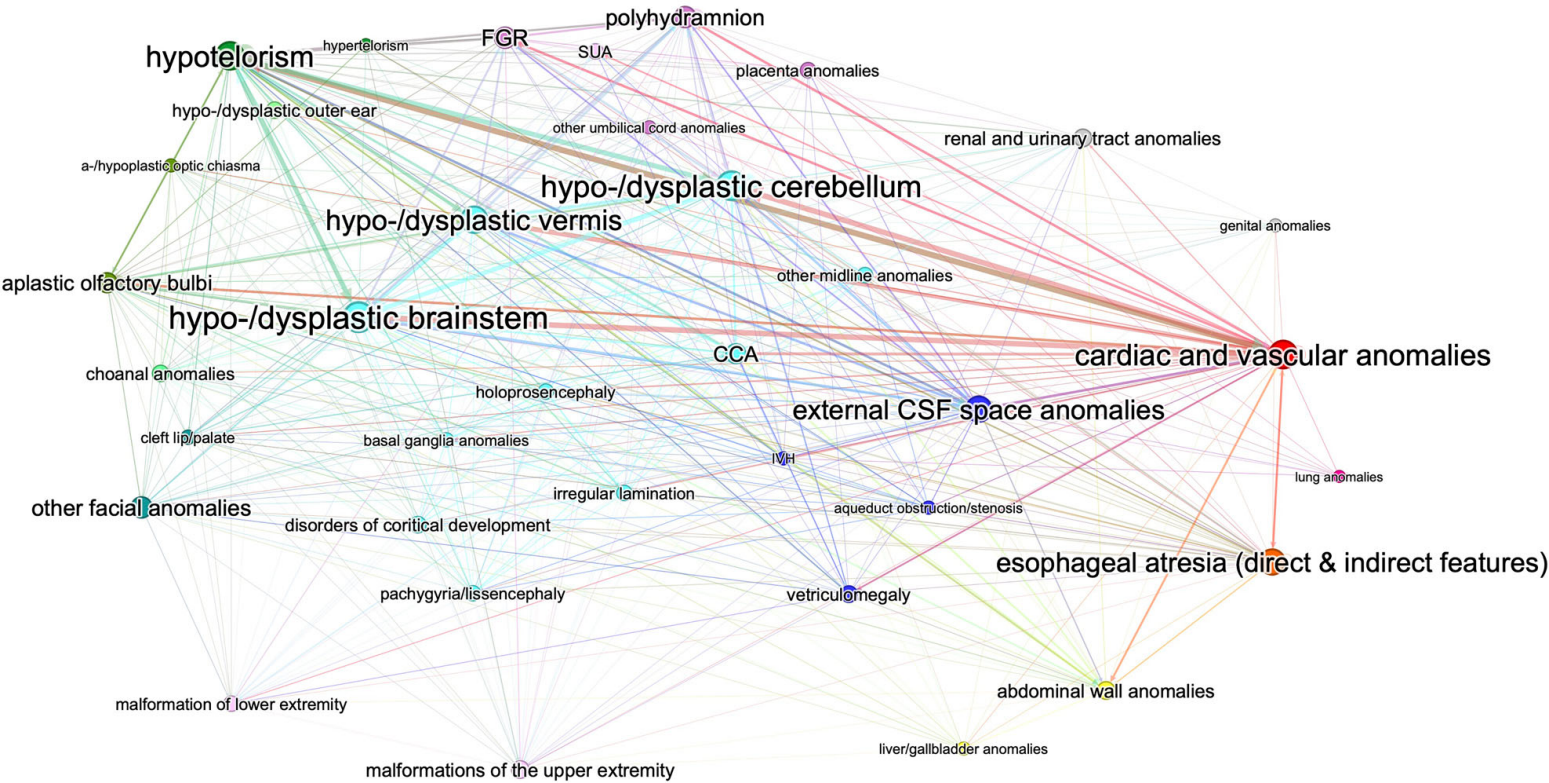
Co-occurrence network of patients with trisomy 18

The co-occurrence networks of fetal MRI morphological features among fetuses with CS (Fig. 3), T13 (Fig. 4), and T18 (Fig. 5) were generated. The assessed features were represented as nodes with larger nodes indicating more frequently observed anomalies. The thickness of each edge/connecting line corresponds to the pairwise frequency of co-occurrence. In addition, color-coding was performed depending on the involved organ system to allow for visual categorization.

Quantitative assessment of the co-occurrence matrices included quantification of edge density (T13: 0.729, T18: 0.705, CS: 0.506), mean clustering coefficient (T13: 0.858, T18: 0.857, CS: 0.826), number of nodes (T13: 43, T18: 36, CS: 29), and number of edges (T13: 1317, T18: 888, CS: 411). Thus, the complexity of the T13 network was the highest despite T18 involving the largest number of patients. The CS network showed the lowest complexity due to the smallest number of patients in this subgroup.

### Morphological disease severity score

Overall, the morphological disease severity scores ranged between 1 and 26. GT13 showed the highest scores (mean = 14.7, SD + /− 6.6, [1–26]), followed by GT18 (mean = 12.1, SD + /− 4.1, [2–21]) and GCS (mean = 11.5, SD + /− 3.9, [5–18]). The disease severity score was associated with gestational age (Pearson correlation coefficient = 0.385, *p* = 0.005). The individual morphological disease severity scores are listed in Table S1.

### Outcome analysis

A detailed overview of outcome data is provided in Supplementary Table S3. Table 5 summarizes the statistical analysis of available outcome data, which was at least partially available in 38 patients. The remaining patients (*n* = 10) were lost to follow-up, and no additional data were available.

**Table 5:** Summary of the statistical analysis of outcome data

		CHARGE	T13	T18
Survival	Sample size	3	0	0
	Disease severity score	9.7 + /− 4.2 (5–13)	n/a	n/a
	Age	8.8 + /− 4.3 (3 years 11 months–12 years)	n/a	n/a
Spontaneous intrauterine fetal demise	Sample size	0	2	6
	Disease severity score	n/a	9 + /− 11.3 (1–17)	12.7 + /− 1.9 (10–15)
	Age (GW)	n/a	20.9 + /− 11.6 (12.7–29.1)	31.8 + /− 11.0 (13.3–41.1)
Postnatal demise	Sample size	0	6	8
	Disease severity score	n/a	16 + /− 8.0 (4–26)	12.3 + /− 5.0 (6–18)
	Age	n/a	0–49 days	0 days–4 years 10 months
Termination of pregnancy	Sample size	2	5	6
	Disease severity score	13.5 + /− 6.4 (9–18)	17.8 + /−3.7 (14–23)	10.7 + /− 5.3 (2–16)
No outcome data available	Sample size	4	3	3

Sample size given as number of patients. Disease severity score and age given as mean +/− standard deviation (range)

*CS* CHARGE syndrome, *T13* trisomy 13, *T18* trisomy 18, *GW* gestational weeks

## Discussion

Using a network approach, a novel definition of the overlapping phenomes of CS, T13 and T18 was established. Among the investigated patient collective, most fetuses were identified as presenting with complex combinations of structural anomalies, consequently harboring an unfavorable outcome prognosis. This is in line with the common clinical presentation of these highly heterogeneous disease entities. However, some fetuses were found to have less severe disease manifestations. To date, many cases with a prenatally proven genetic anomaly of one of the three investigated diseases undergo termination of pregnancy regardless of the severity of morphological manifestations [6, 21]. Only recently, it was suggested that the outcome of some cases may be less adverse in the absence of certain anomalies such as congenital heart disease [4]. In addition, it was shown that even fetuses with cardiac defects might have prolonged life expectancy following postnatal surgery. The proposed disease severity score focuses on the presentation of morphological anomalies – further research with postnatal outcome data will be necessary to assess the applicability of this score regarding clinical disease severity. However, through precise structural assessment and the proposal of this preliminary morphological disease severity score, we hope to enable physicians to counsel their patients more confidently and to increase awareness for future studies investigating the postnatal outcome of children who would have commonly not come to term based purely on their genetic diagnosis.

The detailed results of the phenotypical assessment were summarized during co-occurrence network analysis and calculation of the morphological disease severity score to provide a simplified visual representation of all three complex disease entities in a simple quantitative assessment tool. The combination of observed anomalies was based on the affected organ systems and adjacent anatomical structures. While anomalies of the major organs were summarized for each organ (e.g., CNS) or even combined (e.g., cardiovascular), smaller structures were combined based on their embryological origin: facial anomalies were summarized and differentiated from malformations of the outer ear, inner ear, and eyes based on their varying embryological origin [22, 23].

Regarding the results of the co-occurrence analysis, GT13 presented the most complex network. As T13 is only the second largest group in this collective, this is not purely based on varying sample sizes among the investigated groups, but seemingly due to a more heterogeneous phenotypical presentation of these patients on fetal MRI, particularly affecting the extra-CNS structures.

Comparing these three disease entities and their respective networks, one observation must be emphasized: there is no solitary defining feature prevalent in only one group, highlighting the overlapping phenotypical presentations and necessity for further clinical and genetic assessment. This finding can only be confidently deduced due to the complex morphological assessment and the highly detailed network analysis applied in this study. This phenome-based analysis allowed for more definitive identification of defining, overlapping, and differentiating disease manifestations.

All three groups share complex cardiovascular and CNS anomalies as defining features. Strikingly, the presence of hypotelorism was found to be much more prevalent in fetuses suffering from trisomy 13 or 18 rather than CS, where hypertelorism was more frequently observed (Figs. 3–5)—to our knowledge, these differentiating features are novel findings.

Due to the incomplete postnatal follow-up data of this patient collective, grading of disease severity was based on previously published literature. The highest score (four points) was assigned to CNS anomalies as well as cardiac and vascular defects based on previously published literature indicating poorer survivability and postnatal outcomes. Both cardiac/vascular (e.g., tetralogy of Fallot) and CNS anomalies (e.g., holoprosencephaly) may represent severe structural alterations that require surgical correction or lifelong therapy and care due to profound disabilities [4, 24, 25].

Three points were attributed to the presence of choanal atresia, which requires additional precautions regarding airway management and is associated with more complex malformations, indicating a higher likelihood of unfavorable postnatal outcomes [26, 27].

Most categories included in this proposed morphological disease severity score were rated with two points, including spinal, pulmonary, renal/urinary tract, genital, esophageal/gastric, abdominal wall/bowel, thymic, inner ear, other facial, and placental anomalies, FGR, as well as anomalies of the extremities and oligohydramnios. This grading is based on the potentially major impact on postnatal outcome despite the fact that surgical correction was not always required [14, 28–38].

Last, umbilical cord anomalies and anomalies of the outer ears and eyes were rated with one point, as they are less likely to have a severe impact on long-term survivability compared to the other aforementioned categories [39, 40].

The calculated disease severity scores were surprisingly heterogeneous, ranging from 1 to 26. This heterogeneity may partially be explained by varying gestational ages at fetal MRI. Some fetuses were imaged as early as the late first trimester, which did not allow for assessment of all anatomical details that would have been relevant for the score assessment, but only developed subsequently or would have become visible on MRI at later gestational ages. Similarly, in utero and post-mortem MRI differ in the possibility to assess the intrauterine fluid and the placenta. This is also highlighted in the apparent correlation of disease severity score and gestational age. However, even among fetuses of the same age and with the same underlying genetic disease, gross differences in disease manifestations were observed, once again highlighting the necessity for detailed phenotypical assessment prior to counseling of expectant mothers. In the investigated patient collective, fetuses that succumbed to natural death or termination of pregnancy were found to have higher disease severity scores at prenatal timepoints (mean disease severity score 13.4, SD + /− 5.7, range 2–26)—the majority of them were found to have disease severity scores of ten or higher (26/35 cases).

Due to a lack of complete postnatal follow-up data for all patients investigated in this patient collective, score categories were quantitatively weighted based on the findings of previous studies that assessed individual organ systems and associated outcome data, rather than internal validation of incomplete institutional records of investigated patients. Consequently, the proposed disease severity score must be considered a preliminary tool. Acknowledging the limitations of sample size in this initial study and the lack of complete postnatal follow-up data of this collective, larger prospective studies will be necessary to evaluate long-term outcomes and to correlate prenatal, phenotypical presentation with postnatal survivability. However, the discussed preliminary disease severity score is purely morphological and is a proposed tool to help radiologists and clinicians alike to provide a quantitative estimation of case-based disease burden, rather than relying on genetic testing results alone.

## Supplementary information

ELECTRONIC SUPPLEMENTARY MATERIAL

## Abbreviations

CNS: Central nervous system
CSF: Cerebrospinal fluid
FGR: Fetal growth restriction
GT13: Group trisomy 13
GT18: Group trisomy 18
GW: Gestational weeks
T13: Trisomy 13
T18: Trisomy 18
