# Supplementary material for: Phenotyping trisomies 13 and 18 and CHARGE syndrome in fetal MRI—a proposed phenome-based, morphological disease severity score, and network medicine analysis
Source: Eur Radiol. 2026 Apr 29;36(8):6465–75. doi: 10.1007/s00330-026-12503-w (PMC13342138; doi:10.1007/s00330-026-12503-w)
Supplement: Supplementary file 1 — ELECTRONIC SUPPLEMENTARY MATERIAL [file 330_2026_12503_MOESM1_ESM.pdf]

Diagnosis	Patient ID	in utero/ post mortem	GA at MRI	Sex	Singleton/ gemini	Disease severity score	Optic tracts/ chiasma	Olfactory bulbi	Sulcation & gyration	Lamination	Brain symmetry	Basal ganglia	Holoprosencephaly	Midline structures	Lateral ventricles	Ventricle lining	CSF spaces	Fossa posterior	Intraventricular lesions	Parenchymal lesions	Brainstem	Aqueduct	Cerebellum	Vermis	Skull	Midface anomalies	Chin/ jaw anomalies	Cleft	Tongue	Choanal atresia	Eyes	Inner ear	Outer ear	Vitium cordis	Vascular structures	Lung	Neck	Thymus	Esophagus/ stomach	Liver/ gallbladder	Bowels	Abdominal wall	Kidneys	Urinary tract	Gonads	Upper extremity	Lower extremity	Spine	Umbilical cord	FGR	Placenta	Amniotic fluid
CS	1	iu	26+1	m	s	13	-	+	-	-	-	-	-	+	-	-	-	-	-	-	-	-	-	-	-	-	-	-	-	+	-	+	-	-	+	-	-	-	-	-	-	-	-	-	-	-	-	-	-	-	-	+
CS	1	iu	36+2	m	s	15	-	+	-	-	-	-	-	+	-	-	+	-	-	-	-	-	-	-	-	-	-	-	-	+	-	+	-	-	+	-	+	-	+	-	-	-	-	-	-	-	-	-	-	-	-	+
CS	2	iu	26+0	m	s	12	-	-	-	+	+	-	-	-	-	+	-	+	-	+	+	-	+	-	+	+	-	+	-	-	-	-	+	+	+	-	-	-	-	-	-	-	-	-	-	+	-	-	-	-	-	-
CS	3	iu	21+2	m	s	11	-	+	-	-	-	-	-	-	-	-	-	-	-	-	+	-	+	-	-	-	-	+	-	-	+	-	-	+	-	-	-	-	-	-	-	-	-	-	-	-	-	-	-	-	-	-
CS	3	iu	29+1	m	s	13	-	+	-	-	-	-	-	-	-	-	+	-	-	-	+	-	+	-	-	-	-	+	-	-	+	-	-	+	-	-	+	-	+	-	-	-	-	-	-	-	-	-	-	-	-	-
CS	4	iu	22+5	m	s	5	-	-	-	-	+	-	-	-	-	-	-	-	-	-	-	-	-	-	-	-	+	-	-	-	+	-	-	-	-	-	+	-	-	-	-	-	+	-	-	+	-	-	-	-	-	-
CS	5	iu	25+3	f	s	18	-	+	-	-	-	-	-	-	-	-	-	-	-	+	+	-	+	+	-	+	-	+	+	+	+	-	+	+	-	-	-	+	-	-	-	-	+	-	-	-	-	-	-	-	-	-
CS	5	iu	29+5	f	s	18	-	+	-	-	-	-	-	-	-	-	-	-	-	+	+	-	+	+	-	+	-	+	+	+	+	-	+	+	-	-	-	+	-	-	-	-	+	-	-	-	-	-	-	-	-	-
CS	6	iu	31+3	f	s	11	-	+	-	-	+	-	-	-	-	-	-	-	-	-	+	-	+	+	-	-	-	-	-	-	+	-	-	-	-	-	+	-	+	+	+	-	-	-	-	-	-	-	-	-	-	+
CS*	7	iu	27+0	m	s	8	-	-	-	-	+	-	-	+	+	+	-	-	-	-	-	+	+	+	-	-	-	-	-	-	+	-	-	-	-	-	-	-	+	-	-	-	-	-	-	-	-	-	+	-	-	-
CS*	8	iu	18+2	f	s	9	-	-	+	+	-	-	+	+	+	-	-	-	-	-	+	-	+	+	-	-	-	-	-	-	+	-	+	+	-	-	-	-	-	-	-	-	-	-	-	-	-	-	-	-	-	-
CS*	8	iu	23+1	f	s	9	-	-	+	+	-	-	+	+	+	-	-	-	-	-	+	-	+	+	-	-	-	-	-	-	+	-	+	+	-	-	-	-	-	-	-	-	-	-	-	-	-	-	-	-	-	-
CS**	9	iu	27+6	f	s	8	-	-	-	-	+	-	-	-	+	-	-	-	-	-	+	-	-	-	-	-	-	-	-	-	-	-	-	-	-	-	+	-	+	-	-	-	-	-	-	-	-	-	-	-	+	+
T13	10	iu	20+6	f	s	17	+	+	+	+	+	+	+	+	+	-	-	-	-	-	+	-	+	+	+	+	-	+	-	-	+	-	-	+	-	-	-	-	-	-	-	-	-	-	-	+	-	-	+	-	-	-
T13	11	iu	29+0	m	s	15	+	+	-	-	+	-	-	+	-	-	-	-	-	-	+	-	+	+	-	-	-	+	-	-	+	-	+	+	+	-	-	-	-	-	-	-	+	-	-	+	+	-	-	-	-	-
T13	11	pm	31+3	m	s	15	+	+	-	-	+	-	-	+	-	-	-	-	-	-	+	-	+	+	-	-	-	+	-	-	+	-	+	+	+	-	-	-	-	-	-	-	+	-	-	+	+	-	-	-	-	-
T13	12	iu	34+3	f	g	9	-	-	+	-	-	-	-	-	-	-	-	-	-	-	-	-	-	-	-	-	+	-	-	-	-	-	-	-	-	-	-	-	-	-	-	-	-	-	-	-	-	-	+	+	+	+
T13	13	iu	21+0	f	s	14	-	-	-	-	+	-	-	+	-	-	+	-	-	-	+	-	+	+	+	-	-	-	-	-	+	-	-	+	-	-	-	-	-	-	+	-	-	-	+	+	-	-	-	-	-	-
T13	14	pm	37+5	m	s	18	-	+	+	+	-	+	+	+	+	-	-	-	-	-	+	-	-	-	-	+	-	+	-	+	+	+	-	+	-	-	-	-	-	-	-	-	+	+	-	-	-	-	-	-	-	-
T13	15	pm	24+1	m	s	23	-	+	+	+	+	+	+	-	+	-	-	+	-	-	-	-	+	+	-	-	-	+	-	-	-	+	+	+	-	+	-	+	-	-	-	-	+	-	-	-	+	-	-	+	-	-
T13	16	pm	21+2	f	s	4	-	-	-	-	-	-	-	+	-	-	-	-	-	-	+	-	+	+	-	-	-	-	-	-	-	-	-	-	-	-	-	-	-	-	-	-	-	-	-	-	-	-	-	-	-	-
T13	17	iu	30+0	m	s	9	+	+	+	+	+	+	+	+	+	+	-	+	-	-	+	-	-	-	+	-	-	-	-	-	+	-	-	+	-	-	-	-	-	-	-	-	-	-	-	-	-	-	-	-	-	-
T13	18	iu	26+4	f	s	14	-	+	+	+	-	-	-	-	+	-	+	-	-	-	-	-	+	+	-	+	+	-	-	-	+	-	+	+	-	-	-	-	-	-	-	-	-	+	-	+	+	-	+	-	-	-
T13	19	iu	19+0	f	s	9	+	+	+	+	+	+	+	+	+	-	-	+	-	-	+	-	+	+	-	+	-	+	-	+	-	-	+	-	-	-	+	-	-	-	-	-	-	-	-	-	-	-	-	-	+	-
T13	20	iu	28+6	f	s	19	-	+	+	+	+	+	+	+	+	-	-	+	-	-	+	-	-	+	-	+	-	+	-	+	+	-	+	-	-	-	-	-	+	-	-	+	+	-	-	+	+	-	+	-	-	+
T13	21	iu	28+3	m	s	20	-	+	+	-	+	-	-	+	-	-	+	-	-	-	+	-	+	+	-	+	-	-	-	+	+	-	+	+	-	-	+	+	-	+	-	+	+	-	+	-	-	-	-	-	-	-
T13	22	iu	21+5	m	s	17	+	-	+	+	+	+	+	+	+	-	+	-	-	-	+	+	+	+	+	+	-	-	-	+	+	-	+	-	-	-	-	-	-	-	-	-	+	-	-	+	+	+	+	-	-	-
T13	23	iu	31+2	m	s	20	-	-	-	-	-	-	-	-	-	-	+	-	-	+	-	-	-	-	-	-	+	+	-	-	-	-	+	+	-	-	-	-	+	-	-	-	-	-	-	+	-	-	+	+	+	+
T13	24	iu	13+2	f	s	1	-	-	-	-	-	-	-	-	-	-	-	-	-	-	-	-	-	-	-	-	-	-	-	-	-	-	+	-	-	-	-	-	-	-	-	+	-	-	-	-	-	-	-	-	-	-
T13	25	iu	24+6	m	s	26	-	-	-	+	-	-	-	+	+	+	+	+	+	+	+	+	+	+	+	-	+	-	-	-	+	-	+	+	-	-	-	-	+	+	-	-	+	+	-	-	+	+	+	-	+	-
T18	26	iu	23+3	m	s	11	-	+	-	-	+	-	-	+	-	-	-	+	-	-	+	-	+	+	-	-	-	-	-	-	+	-	-	-	-	-	-	-	+	-	-	-	-	-	-	+	-	-	-	+	+	+
T18	26	pm	25+6	m	s	11	-	+	-	-	-	-	-	-	-	-	+	-	-	-	-	-	-	-	-	-	-	-	-	-	+	-	-	-	-	-	-	-	+	-	-	-	-	-	-	+	-	-	-	+	-	-
T18	27	pm	22+3	f	s	8	-	+	-	-	-	-	-	-	+	-	-	+	-	-	+	-	-	-	-	-	-	-	-	-	-	-	-	+	-	-	-	-	-	-	-	-	-	-	-	-	-	-	-	-	-	-
T18	28	iu	32+1	f	s	13	+	+	+	-	+	-	-	-	-	-	+	-	-	-	+	-	+	+	-	-	+	-	-	-	+	-	-	+	+	-	-	-	-	-	-	-	-	-	-	-	+	-	-	-	-	-
T18	29	pm	40+3	f	s	14	+	+	+	+	-	+	+	+	+	-	-	-	-	-	+	-	-	-	-	+	-	-	-	+	+	-	-	+	-	-	+	-	+	-	+	-	-	-	-	-	-	-	-	-	-	-
T18	30	iu	27+1	f	s	12	-	-	+	-	-	-	-	-	-	-	-	-	-	-	-	-	-	-	+	-	-	-	-	-	-	-	-	+	-	-	-	-	+	+	-	-	-	-	-	-	-	-	-	-	-	+
T18	31	iu	37+2	f	s	11	-	-	-	-	-	-	-	-	-	-	+	-	-	-	+	-	+	-	+	-	-	-	-	-	+	-	+	+	-	-	-	-	-	-	-	-	-	-	-	+	-	-	-	+	+	+
T18	32	iu	33+2	f	s	14	-	-	-	-	-	-	-	-	-	-	+	+	-	-	+	-	+	+	+	-	-	-	-	-	+	-	-	+	-	-	+	-	+	-	-	-	-	-	-	+	-	-	+	+	-	+
T18	33	pm	13+2	m	s	10	-	-	-	-	-	-	-	-	-	-	-	-	-	-	-	-	-	-	-	-	+	-	-	-	-	-	-	+	-	-	-	-	+	-	-	-	+	-	-	-	-	-	-	-	-	-
T18	34	iu	21+0	m	s	18	-	-	-	-	-	-	-	+	-	-	-	+	+	-	+	-	+	+	-	-	+	+	-	+	+	-	+	+	-	-	-	-	+	-	-	+	-	-	-	-	-	-	-	-	-	+
T18	34	pm	33+0	m	s	21	-	+	-	-	-	-	-	+	-	-	-	-	-	-	+	-	+	+	-	+	+	+	-	-	+	-	-	+	-	-	-	-	+	+	-	+	-	+	+	+	-	-	-	-	-	-
T18	35	iu	20+4	m	g	10	-	-	-	-	-	-	-	+	+	-	-	-	-	-	-	-	+	+	-	-	+	+	-	-	-	-	-	-	-	-	-	-	-	-	-	-	-	-	-	-	+	-	-	+	-	+
T18	36	pm	15+0	f	s	8	-	-	-	-	-	-	-	-	-	-	-	-	-	-	+	-	+	-	-	-	-	-	-	-	-	-	-	+	-	-	-	-	-	-	-	-	-	-	-	+	-	-	-	-	-	-
T18	37	iu	26+0	f	s	10	-	-	-	-	-	-	-	-	+	-	-	-	-	-	-	-	+	-	-	-	+	-	-	-	-	-	-	+	-	-	-	-	-	-	-	-	-	-	-	-	-	-	-	-	-	-

Diagnosis	Patient ID	in utero/ post mortem	GA at MRI	Sex	Singleton/ gemini	Disease severity score	Optic tracts/ chiasma	Olfactory bulbi	Sulcation & gyration	Lamination	Brain symmetry	Basal ganglia	Holoprosencephaly	Midline structures	Lateral ventricles	Ventricle lining	CSF spaces	Fossa posterior	Intraventricular lesions	Parenchymal lesions	Brainstem	Aqueduct	Cerebellum	Vermis	Skull	Midface anomalies	Chin/ jaw anomalies	Cleft	Tongue	Choanal atresia	Eyes	Inner ear	Outer ear	Vitium cordis	Extracranial vessels	Lung	Neck	Thymus	Esophagus/ stomach	Liver/ gallbladder	Bowels	Abdominal wall	Kidneys	Urinary tract	Gonads	Hands/arms	Feet/legs	Spine	Umbilical cord	FGR	Placenta	Amniotic fluid
T18	37	pm	28+1	f	s	10	-	-	-	-	-	-	-	-	+	-	-	-	-	-	-	-	-	-	-	-	+	-	-	-	-	-	-	+	-	-	-	-	-	-	-	-	-	-	-	-	-	-	-	-	-	-
T18	38	pm	17+2	f	s	13	-	-	-	-	-	-	-	+	+	-	-	+	-	-	+	-	+	-	-	-	-	-	-	-	+	-	-	+	-	-	-	-	+	+	+	+	-	-	-	-	-	-	-	-	-	-
T18	39	iu	27+5	f	s	12	-	-	+	+	-	-	+	+	+	-	-	-	-	-	+	-	-	+	-	-	-	-	-	-	-	-	-	+	-	-	+	-	+	-	-	-	-	-	-	-	+	-	-	-	-	+
T18	40	iu	23+0	f	s	16	-	+	+	+	+	+	+	+	+	+	+	+	-	-	+	+	+	-	+	-	-	-	-	+	+	-	-	+	-	-	-	-	+	-	-	-	-	-	-	+	-	-	+	-	-	-
T18	40	pm	23+2	f	s	11	-	+	+	+	-	+	+	+	+	+	-	+	-	-	+	+	+	-	+	-	-	-	-	-	+	-	-	+	-	-	-	-	+	-	-	-	-	-	-	-	-	-	-	-	-	-
T18	41	iu	28+5	f	s	7	-	-	-	-	-	-	-	-	-	-	-	-	-	-	-	-	+	+	-	-	-	-	-	-	+	-	-	-	-	-	-	-	-	-	-	-	-	-	-	-	-	-	-	+	-	+
T18	42	pm	12+5	m	s	2	-	-	-	-	-	-	-	-	-	-	-	-	-	-	-	-	-	-	-	-	-	-	-	-	-	-	-	-	-	-	-	-	-	-	-	+	-	-	-	-	-	-	-	-	-	-
T18	43	iu	32+0	f	s	18	-	-	-	-	-	-	-	-	-	-	-	+	-	-	+	-	+	+	+	-	-	-	-	+	+	-	+	+	-	-	+	-	+	-	-	-	+	-	-	-	-	-	-	+	-	+
T18	44	iu	36+3	f	s	13	-	-	-	-	-	-	-	-	-	-	-	+	+	-	+	-	-	+	-	-	-	-	-	-	+	-	-	+	-	+	-	-	-	-	-	-	-	-	-	-	-	-	-	+	+	-
T18	45	pm	17+3	f	s	17	-	-	-	-	-	-	-	+	+	-	-	-	-	-	-	-	-	-	-	+	+	-	-	-	+	-	+	+	-	-	-	-	-	-	-	+	+	-	-	+	+	-	-	-	-	-
T18	46	iu	29+1	m	s	16	-	-	+	-	-	-	-	+	-	-	-	+	-	-	+	-	+	+	-	-	-	-	-	-	+	-	-	+	-	-	-	-	-	-	-	+	+	-	+	+	-	-	+	-	-	-
T18	47	iu	22+3	m	s	15	-	-	-	-	-	-	-	-	+	-	-	-	-	-	+	-	+	+	-	-	-	-	-	+	+	-	-	+	-	-	-	-	-	-	-	-	-	-	-	-	-	-	+	+	+	-
T18	48	iu	19+0	f	g	6	-	-	-	-	-	-	-	-	-	-	-	-	-	-	-	-	-	-	-	-	-	-	-	-	-	-	-	+	-	-	-	-	-	-	-	-	-	-	-	-	-	-	-	+	-	-

Table S1: Summary of all included patients with overview of affected organ systems and disease severity scores

* incomplete genetic testing, mother objected to specific testing for CHARGE syndrome - fetus/child clinically managed as a CHARGE syndrome patient.

** no genetic testing, mother objected genetic testing, fetus/child clinically managed as CHARGE syndrome patient.

CS = CHARGE syndrome, T13 = trisomy 13, T18 = trisomy 18, iu = in utero, pm = post-mortem,

GA = gestational age, m = male, f = female, s = singleton, g = gemini,

+ = pathology, - = no pathology

Region	Anatomical structure	Anomaly	Included in disease severity score
Cranial nerves	Optic tracts/chiasma	aplastic	yes
Cranial nerves	Optic tracts/chiasma	hypoplastic	yes
Cranial nerves	Olfactory bulbi	aplastic	yes
Cranial nerves	Olfactory bulbi	unilateral agenesis	yes
Cortical development	Sulcation & Gyration	irregular	yes
Cortical development	Sulcation & Gyration	pachygyria	yes
Cortical development	Sulcation & Gyration	delayed	yes
Cortical development	Sulcation & Gyration	lissencephaly	yes
Cortical development	Sulcation & Gyration	schizencephalic cleft	yes
Cortical development	Sulcation & Gyration	malrotated hippocampi	no
Cortical development	Sulcation & Gyration	abnormal focal gyration	yes
Cortical development	Sulcation & Gyration	focal cortical dysplasia	yes
Cortical development	Lamination	irregular	yes
Cortical development	Lamination	oedema	yes
Cortical development	Lamination	delayed	yes
Brain/ temporal symmetry	Brain/temporal symmetry	abnormal	no
Brain/ temporal symmetry	Brain/temporal symmetry	too symmetrical	no
Brain/ temporal symmetry	Brain/temporal symmetry	reversed symmetry	no
Holoprosen-cephaly	Basal ganglia	thalami fused	yes
Holoprosen-cephaly	Basal ganglia	agenesis	yes
Holoprosen-cephaly	Holoprosencephaly	semilobar holoprosencephaly	yes
Holoprosen-cephaly	Holoprosencephaly	alobar holoprosencephaly	yes
Holoprosen-cephaly	Holoprosencephaly	lobar holoprosencephaly	yes
Midline structures	Midline structures	corpus callosum agenesis	yes
Midline structures	Midline structures	partial corpus callosum agenesis	yes
Midline structures	Midline structures	hypoplastic corpus callosum	yes
Midline structures	Midline structures	dysplastic corpus callosum	yes
Midline structures	Midline structures	Probst bundles	yes
Midline structures	Midline structures	interhemispheric cyst	yes
Midline structures	Midline structures	falx defect	no
Midline structures	Midline structures	falx agenesis	no

		agenesis of third ventricle	yes
		frontoethmoidal meningoencephalocele	yes
		beaked/deformed tectum	yes
		abnormal pituitary gland	yes
		ectopic pineal gland	yes
		dilated cavum septum pellucidi	no
		fused fornices	yes
CSF spaces	Lateral ventricles	monoventricle	yes
		partial monoventricle	yes
		dilated	yes
		asymmetric but normal size	no
		asymmetric, unilateral dilated	yes
		plexus cysts	no
	Ventricle lining	irregular	yes
		subependymal heterotopia	yes
	CSF spaces	widened	yes
		narrowed	yes
	Fossa posterior	small	yes
		enlarged	yes
		megacisterna magna	no
		retrocerebellar cyst	yes
		dilated sinus	yes
Acquired lesions	Intraventricular lesions	intraventricular haemorrhage	yes
		plexus haemorrhage	yes
	Parenchymal lesions	cerebral oedema	yes
		cerebral haemorrhage	yes
		periventricular cysts	no
		enlarged germinal matrix	yes
Brainstem + aqueduct	Brainstem	hypoplastic	yes
		dysplastic	yes
		kinked	yes
		flattened	yes
		spinally displaced	yes

		fused mesencephalon	yes
		elongated midbrain	yes
	Aqueduct	obstructed/stenosis	yes
Cerebellum + vermis	Cerebellum	hypoplastic	yes
		dysplastic	yes
		partial rhombencephalosynapsis	yes
		rhombencephalosynapsis	yes
		spinally displaced	yes
	Vermis	hypoplastic	yes
		dysplastic	yes
		malrotated	yes
		spinally displaced	yes
Head/skull	Skull	microcephaly	no
		macrocephaly	no
		cebocephaly	yes
		lemon sign	yes
		flat forehead	no
		frontal bossing	no
		forehead oedema	no
		clivus malformation	yes
	Midface anomalies	arhiny	yes
		microrhiny	yes
		flat nose	no
		singular nostril/nasal cavity	yes
		skin appendage at glabella	no
		rudimentary nasal sinus	yes
		rudimentary nose	yes
		agenesis of praemaxilla + teeth	yes
		velum agenesis	yes
	Chin/jaw anomalies	antegnathy	no
		retrognathy	yes
		micrognathy	yes
	Cleft	cleft lip + palate	yes

		cleft palate	yes
	Tongue	glossoptosis	yes
Choanae	Choanal atresia	choanal atresia	yes
		partial agenesis	yes
		hypoplastic	yes
Eyes	Eyes	hypotelorism	yes
		hypertelorism	yes
		microphthalmia	yes
		coloboma	yes
Ears	Inner ear	dysplastic	yes
	Outer ear	low-set	yes
		microtia	yes
		hypoplastic	yes
		dysplastic	yes
Cardiovascular system	Vitium cordis	vitium cordis	yes
	Vascular structures	extracranial vascular anomaly	yes
Lung	Lung	pleural effusions	yes
		hypoplasia	yes
		congenital cystic adenomatoid malformation (CCAM)	yes
Neck	Neck	pouch sign	yes
		cysts at level of thyroid gland	no
		increased nuchal translucency	no
	Thymus	agenesis	yes
Esophagus + organs of the upper quadrants	Oesophagus/stomach	dilated oesophagus (without stenosis)	yes
		oesophageal atresia	yes
		small stomach	yes
	Liver/spleen/gallbladder	dilated gallbladder	yes
		eventration of liver	yes
		elongated right liver lobe	yes
		signal alterations in liver	yes
Bowels + abdominal wall	Bowels	malrotation	yes
		small bowel stenosis	yes
		eventration of bowels	yes

		meconium markings increased (ingestion of blood)	no
	Abdominal wall	omphalocele	yes
		hernia into the cord	yes
Urinary and reproductive systems	Kidneys	double kidney	yes
		horseshoe kidney	yes
		renal cysts	no
		hydronephrosis	yes
		renal agenesis	yes
		enlarged kidneys	yes
		hypoplastic, cystic kidneys	yes
	Urinary tract and suprarenal glands	excentric urinary bladder	no
		disorder of uretrovesicular disjunction	yes
		mass lesion of vesicular wall	yes
	Gonads	micropenis	yes
		hypospadia	yes
		delayed undescended testes	yes
		uterus bicornis	yes
Skeleton	Hands/arms	hexadactyly	yes
		syndactyly	yes
		ectrodactyly	yes
		malpositioned extremities	no
		lobster claw	yes
		enlarged fingers	yes
		adducted thumbs	no
	Feet/legs	rocker-bottom feet	yes
		club feet	yes
		amelia	yes
		malpositioned lower extremity	no
	Spine	lumbar myelomenigocele	yes
		deep lying conus	yes
Placenta + FGR	Umbilical cord	single umbilical artery	yes
		insertio velamentosa	no
		short umbilical cord	yes

	Foetal growth restriction (FGR)	foetal growth restriction (FGR)	yes
	Placenta	inhomogeneous	yes
		oedema	yes
		venous congestion	yes
		haemorrhagic	yes
		lobulated	yes
		thickened	yes
		small	yes
		amniotic band	yes
Amniotic fluid	Amniotic fluid	polyhydramnios	yes
		oligohydramnios	yes

Table S2: Overview of phenotypical assessment and correlating disease severity score

Detailed overview of all observed phenotypical anomalies within this patient collective. Anomalies deemed to be relevant for the long-term outcome prognosis of the patient get awarded with one to three points (depending on the anatomical region and the respective disease severity score category), minor/correctable/irrelevant anomalies did not influence the disease severity score (0 points).

Diagnosis	Patient ID	In utero/ post mortem	GA at MRI	Sex	Singleton/ gemini	Disease severity score	Outcome
CS	1	iu<br>iu	26+1<br>36+2	m	s	13	**alive - 3 years 11 months**:<br>s/p surgical correction of esophageal atresia (Vogt IIIb), s/p fundoplication, s/p tracheostoma for supraglottic obstruction, arrhinencephaly, callosal hypo-/dysplasia, right-sided aortic arch, hypogonadotropic hypogonadism
CS	2	iu	26+0	m	s	12	LTFU
CS	3	iu<br>iu	21+2<br>29+1	m	s	11	**alive - 12 years**:<br>bilateral aplasia of cochlear nerve, recurrent pneumonias, s/p pleurectomy, s/p closure of cleft lip, jaw, palate, s/p surgical correction of esophageal atresia (Vogt IIIb), unilateral paresis of n. recurrens, s/p unilateral partial laser resection of ary-cartilage, s/p surgical correction of unilateral choanal atresia, unilateral tracheomalacia, s/p stent in ductus arteriosus, surgical correction of cardiac defect (not specified), s/p cardiac pacemaker, callosal agenesis, s/p intracerebral hemorrhage, epilepsy
CS	4	iu	22+5	m	s	5	**alive - 10 years 5 months**:<br>s/p bilateral inguinal testis, funicolysis, orchidolysis and orchidopexy, s/p surgical correction of choanal atresia, bilateral iris coloboma, hypoplastic semicircular ducts, Mondini defect of cochlea
CS	5	iu<br>iu	25+3<br>29+5	f	s	18	TOP
CS	6	iu	31+3	f	s	11	LTFU
CS*	7	iu	27+0	m	s	8	LTFU
CS*	8	iu<br>iu	18+2<br>23+1	f	s	9	TOP
CS**	9	iu	27+6	f	s	8	LTFU
T13	10	iu	20+6	f	s	17	TOP
T13	11	iu<br>pm	29+0<br>31+3	m	s	15	TOP
T13	12	iu	34+3	f	g	9	**deceased - 7 weeks**:<br>delayed gyration, PDA, ASD, VSD, s/p necrotizing enterocolitis, perforation of large bowel, and hemicolectomy, Ankyloglossia, bilateral hexadactyly of hands congenital cataract, iris coloboma, facial dysmorphia
T13	13	iu	21+0	f	s	14	TOP
T13	14	iu	37+5	m	s	18	**deceased - 1 day**:<br>large cutaneous and osseous defect at posterior fontanelle, holoprosencephaly, pontine hypoplasia, thickened tectum and tegmentum, arhincephaly, microphthalmia, hypotelorism, cleft lip, jaw and palate, bilateral hexadactyly of hands and feet, single outlet right ventricle, pulmonary atresia, delayed growth/dystrophia, microcephaly, bilateral inguinal testis, incomplete lobulation of lungs, hepatomegaly, unilateral ureter fissus and ureter stenosis, unilateral hydronephrosis, SUA
T13	15	iu	24+1	m	s	23	TOP
T13	16	iu	21+2	f	s	4	**deceased - 0 days (after early induction of labor at 21+0)**:<br>low-set ears, hypertelorism, micrognathia, callosal agenesis, bilateral hexadactyly of hands and feet, omphalocele, VSD, unilateral ureter duplex, uterus bicornis
T13	17	iu	30+0	m	s	9	LTFU

Diagnosis	Patient ID	In utero/ post mortem	GA at MRI	Sex	Singleton/ gemini	Disease severity score	Outcome
T13	18	iu	26+4	f	s	14	LTFU
T13	19	iu	19+0	f	s	9	LTFU
T13	20	iu	28+6	f	s	19	**deceased - 3 days**: craniofacial dysmorphia, omphalocele, bilateral hexadactyly of hands and feet, bilateral rocker-bottom-feet, arhinencephaly, lung hypoplasia, incomplete lobulation of lung, unilateral nephromegaly, unilateral ureter fissus, unilateral adrenal hyperplasia, uterus bicornis, SUA, semilobar holoprosencephaly, cleft lip, jaw and palate
T13	21	iu	28+3	m	s	20	TOP
T13	22	iu	21+5	m	s	17	**IUFD**, stillbirth at 29+1
T13	23	iu	31+2	m	s	20	**deceased - 0 days**: craniofacial dysmorphia, hypertelorism, micrognathia, bilateral hexadactyly of hands and feet, DORV, aortic dextroposition, VSD, ASD, PDA, pulmonary atresia, SUA, median parietal osseous defect of skull, right heart hypertrophy and decompensation
T13	24	iu	13+2	f	s	1	**IUFD**, stillbirth at 12+5
T13	25	iu	24+6	m	s	26	**deceased - 0 days (2 min post partum)**
T18	26	iu<br>pm	23+3<br>25+6	m	s	11	**IUFD**, stillbirth at 25+5
T18	27	iu	22+3	f	s	8	**deceased - 0 days (after early induction of labor at 22+3)**: craniofacial dysmorphia, VSD, right heart hypertrophy, prominent ductus arteriosus, hypoplastic aortic arch, bilateral plexus cysts, subcapsular liver hematoma
T18	28	iu	32+1	f	s	13	**IUFD**, stillbirth (induction of labor at 41+0): hypertelorism, mircoretrognathy, flexion deformity fingers on both hands, common atrium, rudimentary left ventricle, DORV, VSD, AIST, dysphagia lusoria, horseshoe kidneys, microcephaly, unilateral orbitofrontal and periinsular abnormal gyration, arrhinencephaly, interdigitations at medial surface of both hemispheres, lacking gyration of dentate nuclei
T18	29	iu	40+3	f	s	14	**IUFD**, stillbirth at 40+3
T18	30	iu	27+1	f	s	12	LTFU
T18	31	iu	37+2	f	s	11	**deceased - 1 days**: craniofacial dysmorphia, low-set and dysplastic ears, unilateral periauricular appendage, bilateral flexion deformity of fingers, dysplastic nails of hands and feet, diffuse brain edema, subgaleal hematoma, pleural and pericardial effusions, lung hypoplasia, right heart hypertrophy, VSD, tubular AIST, PDA, short sternal bone, elevated diaphragm, ascites, uterus bicornis
T18	32	iu	33+2	f	s	14	LTFU
T18	33	iu	13+2	m	s	10	**IUFD**, stillbirth at 13+2
T18	34	iu<br>pm	21+0<br>33+0	m	s	18	**deceased - 1 day**: craniofacial dysmorphia, dysplastic ears, hypertelorism, bilateral flexion deformity of fingers, esophageal atresia (Vogt IIIb), perimebraneous VSD, PDA, right heart hypertrophy, AIST, enlarged caudate lobe with calcifications, dysplastic kidneys, contracted/narrowed bowels, microcephaly, IVH in lateral and third ventricle
T18	35	iu	20+4	m	g	10	LTFU
T18	36	iu	15+0	f	s	8	TOP

Diagnosis	Patient ID	In utero/ post mortem	GA at MRI	Sex	Singleton/ gemini	Disease severity score	Outcome
T18	37	iu<br>pm	26+0<br>28+1	f	s	10	TOP
T18	38	iu	17+2	f	s	13	**deceased – 0 days (after early induction of labor at 17+2)**:<br>craniofacial dysmorphia, omphalocele, VSD, AIST
T18	39	iu	27+5	f	s	12	TOP
T18	40	iu<br>pm	23+0<br>23+2	f	s	16	TOP
T18	41	iu	28+5	f	s	7	**deceased - 4 years 10 months**: renal insufficiency, VSD, pulmonary hypertension, recurring pneumonias, chronic respiratory insufficiency, hip dislocation, shoulder dislocation, spastic feet, optic hypoplasia, bilateral auditory channel stenosis, pontocerebellar hypoplasia, s/p perinatal intracerebral hemorrhage
T18	42	iu	12+5	m	s	2	TOP
T18	43	iu	32+0	f	s	18	**deceased - 1 week**:<br>bilateral choanal atresia, ASD, VSD, hypoxic ischemic encephalopathy of newborn, esophageal atresia (Vogt IIIb), respiratory insufficiency
T18	44	iu	36+3	f	s	13	**IUFD**, stillbirth at 39+3
T18	45	iu	17+3	f	s	17	**deceased - 0 days (after early induction of labor at 17+3)**: craniofacial dysmorphia, hypertelorism, micrognathia, low-set, dysplastic ears, omphalocele, flexion deformity of upper extremities, VSD, horseshoe kidney, CNS anomalies (not further specified)
T18	46	iu	29+1	m	s	16	TOP
T18	47	iu	22+3	m	s	15	**IUFD**, stillbirth at 30+5:<br>median cleft palate, lung hypoplasia, pleural effusion, VSD, single umbilical artery, hypoplastic placenta
T18	48	iu	19+0	f	g	6	**deceased - 0 days (4 hours post partum)**

Table S3: Summary of available outcome data of investigated fetuses.

* incomplete genetic testing, mother objected to specific testing for CHARGE syndrome - fetus/child clinically managed as a CHARGE syndrome patient.

** no genetic testing, mother objected to genetic testing, fetus/child clinically managed as CHARGE syndrome patient.

AIST = aortic isthmus stenosis, ASD = atrial septal defect, CS = CHARGE syndrome, DORV = double outlet right ventricle, f = female, iu = in utero, IUFD = intrauterine fetal demise, m = male, pm = post-mortem, PDA = persistent ductus arteriosus, s/p = status post, SUA = single umbilical artery, T13 = trisomy 13, T18 = trisomy 18, TOP = termination of pregnancy, VSD = ventricular septal defect
